# Adjunctive therapy for severe malaria: a review and critical appraisal

**DOI:** 10.1186/s12936-018-2195-7

**Published:** 2018-01-24

**Authors:** Rosauro Varo, Valerie M. Crowley, Antonio Sitoe, Lola Madrid, Lena Serghides, Kevin C. Kain, Quique Bassat

**Affiliations:** 1 Centro de Investigação em Saúde de Manhiça, Rua 12, vila da Manhiça, 1929 Maputo, Mozambique; 2 ISGlobal, Barcelona Institute for Global Health, Hospital Clínic, Universitat de Barcelona, Rosselló 132, 5th Floor, 08036 Barcelona, Spain; 3 S. A. Rotman Laboratories, Sandra Rotman Centre for Global Health, University Health Network-Toronto General Hospital, Toronto, Canada; 4 Toronto General Research Institute (TGRI), University Health Network, Toronto, Canada; 5 Women’s College Research Institute, Women’s College Hospital, Toronto, Canada; 6 Department of Immunology and Institute of Medical Sciences, University of Toronto, Toronto, Canada; 7 Department of Medicine, University of Toronto, Toronto, ON Canada; 8 Tropical Diseases Unit, Division of Infectious Diseases, Department of Medicine, UHN-Toronto General Hospital, Toronto, ON Canada; 9 ICREA, Pg. Lluís Companys 23, 08010 Barcelona, Spain; 10 Pediatric Infectious Diseases Unit, Pediatrics Department, Hospital Sant Joan de Déu (University of Barcelona), Barcelona, Spain

**Keywords:** Adjunctive, Treatment, *Plasmodium falciparum*, Malaria, Severe, Cerebral, Experimental, Human, Murine

## Abstract

**Background:**

Despite recent efforts and successes in reducing the malaria burden globally, this infection still accounts for an estimated 212 million clinical cases, 2 million severe malaria cases, and approximately 429,000 deaths annually. Even with the routine use of effective anti-malarial drugs, the case fatality rate for severe malaria remains unacceptably high, with cerebral malaria being one of the most life-threatening complications. Up to one-third of cerebral malaria survivors are left with long-term cognitive and neurological deficits. From a population point of view, the decrease of malaria transmission may jeopardize the development of naturally acquired immunity against the infection, leading to fewer total cases, but potentially an increase in severe cases. The pathophysiology of severe and cerebral malaria is not completely understood, but both parasite and host determinants contribute to its onset and outcomes. Adjunctive therapy, based on modulating the host response to infection, could help to improve the outcomes achieved with specific anti-malarial therapy.

**Results and conclusions:**

In the last decades, several interventions targeting different pathways have been tested. However, none of these strategies have demonstrated clear beneficial effects, and some have shown deleterious outcomes. This review aims to summarize evidence from clinical trials testing different adjunctive therapy for severe and cerebral malaria in humans. It also highlights some preclinical studies which have evaluated novel strategies and other candidate therapeutics that may be evaluated in future clinical trials.

## Background

### The global burden and impact of severe malaria

Malaria is the most important parasitic disease in the world, causing an estimated 212 million infections and 429,000 deaths annually [1]. The greatest burden of severe and fatal disease is borne by children, particularly in sub-Saharan Africa [1]. Humans are unable to develop full immunity to malaria infection. However, acquisition of clinical immunity, which confers protection from life-threatening malaria episodes, is possible but requires repeated exposure to infective mosquito bites. In areas of high transmission, where children are repeatedly exposed to infective mosquito bites from birth, most children will acquire clinical immunity to severe malaria (SM) if they survive their first years of life [2]. In areas of low transmission, however, SM can occur at any age, and is more common among adults, because clinical immunity to malaria takes longer to build, is quick to wane, or simply never occurs. It has been argued that a decrease in the intensity of malaria transmission may put children and adults at risk of severe and fatal disease, precisely as a result of interfering with the natural acquisition of such immune responses [3].

In low-resource settings access to health services is often severely limited, and represents a major constraint to survival for those who develop SM. The case fatality rate (CFR) for SM is heavily dependent on the possibility of reaching the health system, and can range between 20% with in-hospital care, to > 90% when the patient remains at home [4]. It has been estimated that the global annual incidence of SM can be as high as 2 million cases per year [5].

### The pathobiology of severe and cerebral malaria

Both parasite and host determinants contribute to the onset and outcome of severe and cerebral malaria (CM). Host innate immune responses to infection, combined with the sequestration of parasitized erythrocytes (PEs) in the microvasculature of vital organs, such as the brain, result in dysregulated inflammation, endothelial activation, microvascular occlusions, metabolic derangement, and ultimately dysfunction and breakdown of the blood–brain-barrier (BBB) [6]. Sequestered PEs, perfusion abnormalities, haemorrhages, oedema, tissue ischemia, and focal disruptions of the BBB are common fundoscopic and autopsy findings in CM patients and correlate well with disease severity [7–9]. Oxidative stress and axonal injury in the vicinity of brain haemorrhages and in areas of vascular occlusion have also been observed in CM post-mortem studies, and may contribute to neurological dysfunction pre-mortem and in CM survivors [10–12].

There is continued debate within the malaria community as to the utility of animal models and their applicability to human pathophysiology. Notable differences between human CM and *Plasmodium berghei* CM that are generally agreed upon, include the lack of pronounced sequestration of infected red blood cells (iRBCs) and the accumulation of immune cells including leukocytes, monocytes, macrophages, and T cells, in the brains of mice with experimental cerebral malaria (ECM) [13, 14]. In murine models of ECM, intravital microscopy studies have revealed that neurological signs in ECM are associated with vascular leakage and dysfunction of the neuro-immunological BBB, rather than the physiological BBB [15].

 Recently, endothelial protein C receptor (EPCR), a host receptor involved in anticoagulation and endothelial cytoprotection, has been identified as a receptor for *Plasmodium falciparum* erythrocyte membrane protein 1 (PfEMP1) suggesting a link between severe disease and coagulopathy [16]. In addition, dysregulation of the haem-haemopexin axis has been associated with poor clinical outcome and disease severity [17, 18]. Both of these new insights into SM pathogenesis open the door for new therapeutic options.

### Primary treatment of severe and cerebral malaria

Severe malaria is a complex multi-system disease that may be differently defined according to the age group it affects, as clinical manifestations may vary between adults and children. However, it should be noted that the differences between those age groups might not be due to disparities in pathology but due to the under-recognition of complications in young children with SM, as is the case for acute kidney injury [19]. For epidemiological purposes, SM can be defined as the confirmation of a malarial infection in the presence of one or more of a series of syndromes or conditions, including impaired consciousness, acidosis, hyperlactataemia, hypoglycaemia, severe anaemia, acute kidney injury, jaundice, pulmonary oedema, significant bleeding, hyperparasitaemia, or shock [5]. CM, perhaps the most feared complication of malaria, is characterized by severe impairment of consciousness (deep coma) in the absence of other alternative explanations or diagnoses. Impaired consciousness, together with severe respiratory distress, has one of the highest mortality rates of the severe complications [5]. Beyond impaired consciousness, CM can also present with repeated seizures or other neurological abnormalities. CM is associated with long-term cognitive and neurological deficits in up to one-third of survivors, including hemiparesis, cerebellar ataxia, cortical blindness, hypotonia, spasticity, aphasia, seizure disorders, behavioural disorders, and attention-deficit hyperactivity disorder (ADHD) [20–25].

*Plasmodium falciparum* is responsible for the majority of malaria-associated morbidity and mortality. In the absence of prompt and effective treatment, *P. falciparum* infection may progress to severe and potentially fatal forms. Parenteral artesunate is now widely accepted as the standard of care for the treatment of SM, both in adults and children, following the landmark SEAQUAMAT and AQUAMAT trials that demonstrated its superiority over quinine [26, 27]. Recently, intramuscular artesunate administration has proven to be non-inferior to intravenous artesunate in reducing parasitaemia ≥ 99% at 24 h in children with SM [28]. However, even with the improved efficacy of artesunate, CFR for SM (8.5% in children and 15% in adults) and in particular CM (18 and 30%, respectively) remain high [26, 27]. Therefore, treatment with potent artemisinin-derivatives alone is insufficient to prevent death or neurological disability in all patients with SM. Adjunctive therapy, based on modulating host response to infection, could reduce malaria-associated morbidity, mortality and could enhance and extend the clinical utility of current anti-malarials. General declines of malaria and SM burden, decreases in the CFR for malaria and difficulties in detecting reductions in mortality rates may hinder the evaluation of those interventions due to the need of recruiting large numbers of patients [29]. In this respect, it is necessary to creatively innovate in the design of clinical trials with more precise sample sizes, more accurate clinical predictors and surrogate endpoints for mortality like plasma lactate concentration [29–31]. It is recommended that patients with SM with signs of serious bacterial infection receive intravenous antibiotics [5]. However, their effect on mortality and/or clinical outcome have not been tested in any randomized controlled trial (RCT).

### The role of adjunctive therapy in severe malaria treatment

The host immune response plays a central role in the onset, severity and outcome of malaria infections and this has promoted the search for immunomodulatory adjunctive therapy to improve clinical outcome. Adjunctive therapy is used in combination with primary anti-malarial treatment, with the aim of improving efficacy, or reducing disease-associated complications. To date, several types of putative adjunctive therapy have been tested in SM without success. Malaria immunopathogenesis is complex and targeting a single pathway may be insufficient to reduce mortality or improve neurological outcomes. Targeting multiple pathways, either by the use of multiple interventions (which is more complicated to deliver and increases the risk of adverse events, drug interactions and costs), or alternatively, by using a single intervention that targets multiple pathways implicated in the pathobiology of SM, could potentially lead to improved outcomes. Effective adjunctive therapy must be safe, have a clear benefit over anti-malarial use alone, be effective as a late-stage intervention, be minimally invasive, inexpensive, and ideally feasible to implement in low-resource endemic settings, where the bulk of SM occurs. The objective of adjunctive therapy should be the improvement of clinical outcome, and/or reduction of mortality, in addition if possible of the prevention of long-term neurocognitive deficits. This review aims to summarize recent evidence highlighting various approaches currently being pursued as adjunctive therapy for SM and CM. The review will focus on therapy tested in humans in RCTs, and it will also mention some preclinical studies that have evaluated some novel strategies and candidate therapeutics that may be evaluated in future clinical trials.

### Search methodology

RCTs were identified through electronic searches of PubMed without any language or date restrictions and limited to humans. PubMed was searched (accessed 15 June 2017) through the use of a broad sensitive filter using following combinations: “malaria AND adjunctive therapy” (124 results), and “severe malaria AND adjunctive therapy” (81 results) and “cerebral malaria AND adjunctive therapy” (61 results). The references of the retrieved papers were used to search for additional studies. Adjunctive therapy assessed in RCTs is summarized in Table 1. These RCTs cover a period of 33 years (from 1982 to 2015). Thirty-two RCTs were included in the Table. RCTs that did not report data on clinical outcomes or those performed in patients without severe or cerebral malaria were excluded. In the text, some studies performed in uncomplicated malaria are discussed. Clinicaltrials.gov was also searched for ongoing RCTs or completed RCTs with no published data. To identify relevant preclinical models PubMed was searched (accessed 15 June 2017) using the following search terms: “experimental cerebral malaria” (453 results) and “experimental cerebral malaria AND adjunctive therapy” (21 results). Studies were included if they were published after 2010, peripheral parasitaemia at time of adjunctive therapy administration was more than 5%, and the intervention had a benefit after the onset of symptoms.

**Table 1:** List of randomized controlled trials of adjunctive therapy in severe malaria

Author, year, country, references	Antimalarial	Adjuvant therapy	Dosage and route	Study design	Type of malaria	Ages	Sample size	Outcome
Immunomodulation								
Warrell et al. 1982, Thailand, [31]	IV quinine	Dexamethasone	IV; children 0.6 mg/kg at the start followed by 7 doses of 0.2 mg/kg at 6-h intervals; adults 0.5 mg/kg at the start followed by 7 doses of 10 mg each (total duration of treatment 48 h)	RCT, DB, PC	CM	6–70 years	100	Failed to decrease mortality. Increased risk of adverse events (prolonged coma, pneumonia and gastrointestinal bleeding)
Hoffman et al. 1988, Indonesia, [32]	IV quinine	Dexamethasone	IV; initial dose, 3 mg/kg; total, 11.4 mg/kg per 48 h	RCT, DB, PC	CM	1.5–42 years	38	No differences in mortality, parasite and fever clearance times or incidence of complications
Taylor et al. 1992, Malawi, [34]	IV quinine	Immunoglobulin (IFAT antimalarial Ab)	IV; 400 mg/kg over 3 h	RCT, DB, PC	Coma	1–12 years	31	Increased mortality but not statistically significant. No differences in parasite and fever clearance times or incidence of complications
Havlik et al. 2005, Thailand, [36]	IV artesunate	Curdlan sulphate	IV; 4 mg/kg over 30 min/8 h (adjusted dose according to APTT)	RCT, DB, PC	SM but not CM (Phase IIB); SM and CM (Phase IIC)	12–60 years	Phase IIB: 44; Phase IIC: 26	No differences in mortality or parasite clearance times. Trend to improve duration of coma and fever clearance time
van Hensbroek et al. 1996, The Gambia, [37]	IM quinine and IM artemether ± oral pyrimethaminesulfadoxine	anti-TNF mAb	IV; 5 mg/kg over 15 min	RCT, DB, PC	CM	1–9 years	624	No differences in mortality, coma recovery or complications. Lower fever clearance time. Trend towards faster parasite clearance time. Higher rate of neurological sequelae
Di Perri et al. 1995, Burundi, [38]	IV quinine	Pentoxifyline	IV; 10 mg/kg/24, 72 h	RCT	CM	< 14 years	56	Lower mortality not statistically significant. Significant reduction in coma recovery
Das et al. 2003, India, [39]	IV quinine	Pentoxifylline	IV; 10 mg/kg/24, 72 h	RCT	CM	> 18 years	52	Improved mortality, not statistically significant. Significant reduction in coma recovery time
Hemmer et al. 1997, Germany, [40]	1. IV quinine + doxycycline; 2. oral mefloquine or halofantrine	Pentoxifylline	IV; 5 mg/kg/24 h for 5 days	RCT, DB, PC	UM and CM	22–69 years	51	No differences in mortality, clinical outcomes or laboratory parameters. More side effects
Looareesewam et al. 1998, Thailand, [41]	IV artesunate	Pentoxifylline	IV; low (0.83 mg/kg/h) or high (1.67 mg/kg/h) over 72 h	RCT, DB, PC	SM	16–60 years	45	No significant differences in fever and parasite clearance time or in clinical outcomes
Lell et al. 2005, Kenya, [42]	IV quinine	Pentoxifylline	IV; 10 mg/kg/24 h for 72 h	RCT, DB, PC	CM	9 month–8 years	15	Higher mortality. No difference in coma recovery, incidence of complications or neurological sequelae. Trend to faster fever and parasite clearance times
Decreasing procoagulant effects								
Hemmer et al. 1991, Germany, [52]	1. IV quinine + oral doxycycline or oral mefloquine; 2. IV quinine + oral doxycycline	Heparin or acetylsalicylic acid (ASA)	IV: Heparin 70 U/kg/day SC. for 5 days; ASA 500 mg on days 0, 2, 4	RCT	SM	> 14 years	97	No difference in fever, parasite clearance, or time to discharge
Decreasing cytoadherence and sequestration								
Maude et al. 2014, Bangladesh, [57]	IV artesunate	Levamisole	Oral, 150 mg, single dose	RCT, OL	SM	21–45 years	56	No differences in mortality, parasite clearance time, ‘sequestration ratio’ or normalization of plasma lactate
Improving liver function								
Treeprasertsuk et al. 2009, Thailand, [80]	IV artesunate	Ursodeoxycholic acid	IV; 750 mg/day, 2 weeks	RCT, DB, PC	SM with jaundice	> 15 years	80	Safe, but no differences between liver test, fever and parasite clearance times
Restricting iron availability								
Gordeauk et al. 1992, Zambia, [81]	IV quinine +oral pyrimethaminesulfadoxine	Deferoxamine	IV; 100 mg/kg/day over 72 h	RCT, DB, PC	CM	20–54 months	83	Lower mortality, not statistically significant. Faster coma recovery time and parasite clearance time
Thuma et al. 1998, Zambia, [82]	IV quinine	Deferoxamine	IV; 100 mg/kg/day over 72 h	RCT, PC	CM	< 6 years	352	Non-significant trend to faster recovery from coma. No statistical differences in mortality
Mohanty et al. 2002, India, [83]	IV quinine and oral doxycycline	Deferiprone	Oral; 75 mg/kg/day in 12 hourly divided doses over 10 days	RCT, DB, PC	SM	13–84 years	45	Faster fever, parasite clearance and coma recovery time. No differences in mortality
Prevention of seizures								
White et al. 1988, Thailand, [85]	IV quinine	Phenobarbital	IM; 3.5 mg/kg, single dose	RCT, DB, PC	CM	6–78 years	48	Fewer convulsions
Crawley et al. 2000, Kenya, [86]	IV quinine	Phenobarbital	IM; 20 mg/kg, single dose	RCT, DB, PC	CM	19–65 months	340	Fewer convulsions. Higher mortality
Decreasing intracranial pressure								
Namutangula et al. 2007, Uganda, [91]	IV quinine	Mannitol	IV; 1 g/kg	RCT, DBO, PC	CM	6–60 months	156	Did not significantly reduce time taken to regain consciousness, sit unsupported, or mortality
Mohanty et al. 2011, India, [92]		Mannitol	IV; 1.5 g/kg over 15 min, followed by 0.5 g/kg every 8 h until the patient regained consciousness or for a maximum period of 72 h	RCT, OL, PC	CM with brain swelling	25–31 years	61	Trend towards higher mortality in mannitol group. Mannitol prolonged coma recovery
Fluid resuscitation								
Maitland et al. 2005, Kenya, [97]	IV quinine	Human albumin/saline	IV; 20 mL/kg of either 4.5% human albumin solution or 0.9% saline vs control (fluids maintenance group)	RCT, OL	SM with either moderate and severe acidosis	> 1 years	150	Safe and resulted in significantly lower mortality. Acidosis did not improve
Akech et al. 2006, Kenya, [98]	IV quinine	Human albumin/gelofusine	IV; 20–40 mL/kg of either 4.5% human albumin solution or gelofusine	RCT, OL	SM	> 3 years	88	Trend to lower mortality, not statistically significant with albumin. No difference between shock and acidosis recovery. Higher neurological sequelae with albumin group
Fluid resuscitation								
Maitland et al. 2011, Uganda, Kenya, Tanzania, [99]	IV quinine	Human albumin/saline	20 mL/kg of either 4.5% human albumin solution or 0.9% saline vs (fluids maintenance group)	RCT, OL	SM	2 month–12 years	1793 SM cases out of 3123 total sample size	Higher mortality in children treated with bolus
Decreasing oxidative stress								
Watt et al. 2002, Thailand, [105]	IV artesunate	N-Acetylcysteine	IV; 300 mg/kg over 20 h	RCT, DB, PC	SM	> 18 years	30	Faster normalization of lactate levels and Glasgow Coma Score
Treeprasertsuk et al. 2003, Thailand, [106]	IV artesunate	N-Acetylcysteine	IV, oral: 3 different regimes	RCT, PC	SM	14–16 years	108	No differences in mortality, fever and parasite clearance time. No differences in adverse events between groups
Charunwatthana et al. 2009, Bangladesh, Thailand, [107]	IV artesunate	N-Acetylcysteine	IV; 300 mg/kg over 20 h	RCT, DB, PC	SM	30–39 years	108	No differences in clearance of elevated plasma lactate levels, coma recovery times, mortality, fever clearance time, and complications or adverse events
Correcting lactic acidosis								
Khrisna et al. 1994, Thailand, [112]	IV quinine	Dichloroacetate	IV; 46 mg/kg, single dose	not stated	SM	> 14 years	45	Decreased lactate concentrations. No evidence of toxicity. Mortality, incidence of complications and clinical/parasitological measures of recovery did not differ
Correcting lactic acidosis								
Khrisna et al. 1995, Ghana, [113]	IM quinine	Dichloroacetate	IV; 50 mg/kg, single dose	RCT, OL, PC	SM	1.5–12 years	18	Decreased lactate concentrations. No differences in mortality, fever or parasite clearance times
Khrisna et al. 1996, Thailand, [114]	IV quinine	Dichloroacetate	IV; 46 mg/kg single dose	RCT, OL, PC	SM	> 14 years	20	No differences in mortality, greater decrease in lactate concentrations
Agbenyega et al. 2003, Ghana, [115]	IV quinine	Dichloroacetate	IV; 50 mg/kg, single dose	RCT, DB, PC	SM	1–12 years	124	Significantly reduced the concentration of blood lactate
Increasing NO availability								
Hawkes et al. 2015, Uganda, [119]	IV artesunate	Nitric Oxide	inhaled, 80 ppm	RCT, B, PC	SM	1–10 years	180	No differences in levels of Ang-2. No differences in mortality, recovery rates or parasite clearance time
Mwanga-Amumpaire et al. 2015, Uganda, [120]	IV artesunate	Nitric Oxide	inhaled, 80 ppm	RCT, OL, PC	CM	2 month–2 years	92	Did not increase Ang-1, did not reduce mortality rate. Similar clinical outcomes and neurological sequelae between groups

*Ab* antibody, *Ang* angiopoeitin, *APPT* activated partial thromboplastin time, *CM* cerebral malaria, *IM* intramuscular, *IV* intravenous, *mAb* monoclonal antibody, *MO*: months, *NO* nitric oxide, *OL* open-label, *PC* placebo-controled, *PPM* parts per million, *SC* subcutaneous, *SM* severe malaria, *UM* uncomplicated malaria, *YR* years

### Adjunctive therapy for the treatment of severe and cerebral malaria in humans

#### Immunomodulation

Based on the critical role of the host response in determining the onset, severity and outcome of *P. falciparum* infection, different adjunctive therapy has been evaluated to modify this pathophysiological pathway.

##### Corticosteroids

With the aim of reducing swelling and inflammation in the brain, corticosteroids were one of the first treatments proposed as an adjunctive therapy for SM based on successful case reports. However, dexamethasone failed to demonstrate a decrease in mortality in two clinical trials testing different doses in adults with SM, although the small sample sizes and lack of power do not allow ruling out a clear effect on mortality [32–34]. Furthermore, one of the studies showed an increased risk of adverse events (prolonged coma, pneumonia and gastrointestinal bleeding) within the dexamethasone group compared to those receiving placebo [32]. No additional RCT have tested corticosteroids in SM, and the use of dexamethasone is currently not recommended in its management.

##### Intravenous immunoglobulin

Similarly to what occurred with corticosteroids, treatment with intravenous immunoglobulin was associated with increased deleterious outcomes compared to the placebo group, including higher mortality and more neurological *sequelae* in children [35]. The clinical failure of this therapy may reflect the lack of success to reverse cytoadherence and sequestration [35].

##### Curdlan sulfate

Curdlan sulfate (CS), a sulfated 1 → 3-β-d glucan, previously shown to be a potent human immunodeficiency virus (HIV) entry inhibitor, and known to inhibit *P. falciparum* in vitro, has been tested in two RCTs due to its capacity to modulate the immune response to *P. falciparum* [36]. As a sulfated polysaccharide (similar to heparin), CS would be expected to have some anticoagulant properties, and confer certain direct and non-specific effect on cytoadhesion and rosetting. Neither of the studies demonstrated differences in mortality, possibly on account of small sample sizes, but CS was safe and appeared to reduce the severity of the disease process [37].

##### Anti-TNF therapy

Therapy targeting tumour necrosis factor (TNF) and its effects have also been explored. Two different strategies have been evaluated in RCTs. One trial used monoclonal antibodies to inhibit TNF function. No difference in mortality was shown and moreover, there was an increased risk of neurological *sequelae* in the experimental group [38]. The retention of TNF by the antibody within the circulation may explain this deleterious effect [38]. Pentoxifylline (PTX), a phosphodiesterase inhibitor, can reduce levels of TNF and has been tested in different studies with controversial results. Two studies showed an improvement in survival and a significant reduction in coma recovery time [39, 40]. However, three others studies comparing adjunctive PTX treatment to placebo showed no clinical benefit [41–43]. One of the studies also showed higher than expected mortality rates [43]. Taking into account these data and the small samples of the studies, there is no clear evidence to propose PTX as an adjunctive therapy.

##### Charcoal

Oral activated charcoal (oAC) can modify the immune response against malaria infection. In a study with ECM, oAC demonstrated a significant reduction in pro-inflammatory cytokines and improvement in survival [44]. Furthermore, oAC was safe and well tolerated in humans in a Phase I trial and did not interfere with the pharmacokinetics of parenteral artesunate [44]. A RCT in children with uncomplicated malaria to assess safety and parasite clearance times of oAC in combination with intravenous artesunate has finished in Mali but results are yet to be published (NCT01955382), and no trials including patients with SM have been conducted. Importantly, the route of this intervention, similarly to what occurs with oral medications, may prove to be a further hindrance, as critically ill children are unable to swallow and the use of nasogastric tubes may prove difficult.

##### PPAR-gamma agonists

Peroxisome proliferator-activated receptor-γ (PPAR-γ) agonists are attractive adjunctive candidates as they modulate multiple pathways implicated in the pathobiology of SM by reducing excessive inflammation and neurovascular leak, and by enhancing neuroprotective and anti-oxidant mechanisms [45–48]. Rosiglitazone modulates the innate host immune response to malaria [49]. In a murine model of ECM, this drug showed specific benefits by improving survival and reducing neurological impairments [48]. In a RCT in young adults with uncomplicated malaria, rosiglitazone was safe and well tolerated and those receiving rosiglitazone had lower levels of pro-inflammatory biomarkers and faster parasite clearance times [50]. Those patients receiving rosiglitazone also had increased levels of the considered “protective” brain-derived neurotrophic factor (BDNF) and reduced endothelial activation [48, 50]. A phase IIa trial to prove safety and tolerability of rosiglitazone in children under 12 years of age has recently concluded in Mozambique demonstrating the safety and good tolerability of rosiglitazone in children with uncomplicated malaria [51]. Furthermore, a phase IIb trial is now ongoing at the same site to test the efficacy of rosiglitazone as adjuvant therapy to intravenous artesunate for improving clinical and neurological effects of SM (NCT02694874).

#### Decreasing procoagulant effects

As SM induces a procoagulant state [52], different drugs with anticoagulant potential (in addition to curdlan sulfate, already mentioned in a previous paragraph) have been studied as adjunctive therapy. A prospective randomized study in adults with uncomplicated and severe falciparum malaria examined acetylsalicylic acid and low-dose heparin [53]. Neither of these treatments showed beneficial effect on clinical, haemostatic or parasitic parameters. Sulfated glycosaminoglycans (GAG), including heparin and sevuparin, can disrupt rosette formation and inhibit cytoadherence to endothelial cells, and have been proposed as potential adjunctive therapy [54, 55]. However, only one study examined their effects in a RCT. Sevuparin sodium, a heparan sulfate mimetic, was tested in adults with uncomplicated malaria to determine its tolerability and pharmacokinetics when administered as an intravenous infusion in combination with atovaquone–proguanil, proving to be well tolerated [56]. Sevuparin reduced merozoite invasion as the mean relative number of ring iRBCs was lower in the experimental group vs the control group and the treatment resulted in the desequestration of RBC infected with mature parasites as more of these were detected in peripheral circulation [56].

#### Decreasing cytoadherence and sequestration

Levamisole is a specific alkaline-phosphatase inhibitor mainly used to treat intestinal helminths. It was suggested as an adjunctive therapy candidate after showing its capacity to decrease iRBC sequestration in falciparum malaria in vivo [57]. However, a RCT in Bangladesh, which explored the effect of a single levamisole hydrochloride dose (oral, 150 mg, single dose) in adult patients with SM showed no benefit compared to placebo when administered as adjuvant to intravenous artesunate [58]. As in other studies in which intravenous artesunate is used, its fast effect in killing *P. falciparum* parasites may have blurred the benefits of the adjuvant therapy.

#### Reduction of parasite biomass

Exchange blood transfusions (EBT) and erythrocytapheresis have been used as an adjunctive treatment in SM based on the hypothesis that infusing fresh whole blood or uninfected erythrocytes resulting in replenishing erythrocytes lost to parasitization, and reducing iron and other toxic bioproducts associated with infection, could lead to improved outcomes in patients with very high parasitaemia. To date, no prospective RCT of EBT or erythrocytapheresis has been conducted, and despite their frequent use these interventions remain controversial. Numerous case reports and retrospective studies have been conducted but there is limited evidence that such approaches improve parasite clearance times or enhance survival in artesunate-treated patients [59–67]. EBT and erythrocytapheresis may be options in high-resource settings with cases of imported malaria, although current expert opinion tends not to recommend them as adjuvant therapy [68–70]. Such approaches, however, are unfeasible in resource-constrained settings and in communities where the prevalence of HIV and other blood-borne transmissible diseases is high.

#### Improving anaemia and liver function

Severe malarial anaemia (SMA) is an important syndrome of SM and is associated with increased clearance of infected and non-infected erythrocytes and dysregulated haematopoiesis. Blood transfusions are not routinely recommended as a treatment for SMA [71–73]. Erythropoietin has immunomodulation effects and has been shown to reduce clinical signs of ECM in murine models, possibly in relation to its capacity to reduce neural hypoxia and cerebral pathology [74, 75]. In murine ECM models, erythropoietin co-administered with artesunate was associated with an improvement in clinical recovery and global survival rates [76]. In an open-labelled study in children with CM, erythropoietin was safe and well tolerated when administered with quinine [77]. A randomized trial of recombinant human erythropoietin (rHuEPO) in children with CM was prematurely stopped in Mali (EPOMAL Study; ClinicalTrials.gov Identifier: NCT00697164), although preliminary data demonstrated the short-term safety of high doses of erythropoietin (1500 U/kg/day rHuEPO) administered for 3 days (NCT00697164, unpublished data, Picot S, pers. comm.).

Malaria-associated liver injury, including unconjugated hyperbilirubinemia, intrahepatic cholestasis, elevated serum aspartate (AST) and alanine aminotransferase (ALT) levels, and jaundice is not uncommon [78–80]. These symptoms often indicate severe illness and are associated with a higher incidence of complications in a malaria infection [80]. Ursodeoxycholic acid (UDCA) is used in the treatment of cholestatic liver disease and was tested as an adjunctive therapy in adult patients with SM and jaundice with the intention of improving liver function [81]. Although UDCA proved to be safe, it did not significantly improve liver tests. Severity of hyperbilirubinemia, concomitant co-infections and early treatment with intravenous artesunate may explain these results [81].

#### Restricting iron availability

Iron chelators such as desferrioxamine (DFO) or deferiprone were proposed as adjunctive therapy for malaria. As malaria parasites require iron to multiply, reducing the availability of iron could inhibit parasite replication, with the caveat that these agents could contribute to or exacerbate anaemia. A number of small RCTs, not powered to assess mortality, have evaluated the use of iron chelators in SM, showing a tendency to reduce coma and achieve faster parasite clearance times [82–84]. However, data remain insufficient to support the use of iron chelators in the treatment of SM [85].

#### Prevention of seizures

In CM, seizures are usually associated with a higher mortality and a higher risk of neurological *sequelae* [5]. Based on this reasoning, anticonvulsants have been used to prevent seizures in CM. A first RCT, conducted in children, demonstrated that a single intramuscular injection of phenobarbitone (3.5 mg/kg) could reduce the incidence of convulsions, although it did not improve mortality [86]. A subsequent RCT in Kenya in 340 children with CM [87], showed that a single prophylactic intramuscular dose of phenobarbital (20 mg/kg) could reduce the frequency of seizures compared to children receiving placebo. However, mortality was doubled in the group receiving phenobarbital. Respiratory depression caused by phenobarbital and is interaction with other intravenous anticonvulsants could explain this negative effect [84]. Consequently, seizure prophylaxis with phenobarbital could not be recommended as adjunctive therapy for CM and others trials with appropriate design, bigger sample and distinct anticonvulsant doses are required [88]. A recent study in Malawi, assessing the effect of enteral levetiracetam vs phenobarbital to control acute seizures in children with CM has recently finished demonstrating that levetiracetam appeared to have a better safety profile than phenobarbital and a similar effect in the control of neurological complications and mortality (NCT01660672, unpublished data, Birbeck GL, pers. comm.).

#### Decreasing intracranial pressure

Recent studies using magnetic resonance imaging (MRI) in paediatric patients from Malawi demonstrated that children that died from CM had increased cerebral swelling, as compared to those who survived [89]. In fatal cases cerebral swelling progresses to respiratory arrest prior to death. A neuroimaging study in adult and paediatric patients with CM from India showed that both groups had traits characteristic of posterior reversible encephalopathy syndrome [90]. In a RCT in Kenya, mannitol adjunctive therapy controlled intermediate intracranial hypertension but could not prevent the development of intractable intracranial hypertension and did not affect mortality in children with CM [91]. An Ugandan RCT showed that one dose of mannitol had no adverse effects but also no impact on clinical outcomes or mortality in children with SM [92]. More recently, a computed tomography (CT) study demonstrated that brain swelling is a common finding in adults with CM, although brain swelling did not correlate with coma depth or survival [93]. In the same study, patients were randomized to receive either mannitol or placebo. The group receiving mannitol showed a longer coma duration and higher mortality [93]. A limited understanding of the pathogenic mechanism leading to increase brain swelling, inadequate doses of mannitol and small sample sizes may explain these results. In light of these findings, mannitol cannot be recommended as adjunctive treatment for malaria.

#### Fluid resuscitation

Appropriate fluid management in cases of SM has been controversial and there is no conclusive evidence to guide fluid management [73, 94]. While some studies have proposed an important role for impaired tissue perfusion in the outcomes of SM [95, 96], others have argued that hypovolemia does not occur in cases of severe and moderate malaria [97]. Some studies have explored the effects of fluid infusion in SM patients, and showed that fluid resuscitation with albumin compared with saline and gelofusine may reduce mortality [98, 99]. Recently, a large RCT (FEAST trial) was conducted in six different centres in Africa to compare volume expansion with boluses of albumin or saline to standard maintenance fluids in severely ill children [100]. The study was stopped because of higher mortality in the intervention groups. Fifty-seven per cent of those children had SM (1793 out of 3123 patients) and results in the malaria-confirmed cases were consistent with the larger group [100]. Excess of mortality seemed to be related to refractory shock rather than fluid overload in the boluses groups [101, 102]. Current recommendations indicate the need to individually assess the volume status of each patient to guide treatment, a general contra-indication for colloids, and in children a recommendation to avoid bolus fluids even in case of moderate hypotension and severe dehydration or metabolic acidosis [5].

#### Decreasing oxidative stress

Severe malaria is associated with oxidative stress that may be harmful due to the damaging effects of free radicals on cells, increased erythrocyte rigidity and impaired microcirculatory flow [103, 104]. N-acetylcysteine (NAC) is a widely used anti-oxidant that scavenges free radicals, and can reduce expression of endothelial ligands in SM [105]. The use of NAC as adjunctive agent to reduce the negative aspects of oxidative stress associated with SM infection has been investigated. A pilot study in Thailand demonstrated a shorter time in normalization of lactate levels and Glasgow Coma Score with NAC [106]. A RCT in 108 adults with SM showed NAC to be safe and well tolerated, but to have no effect on clinical outcomes or mortality [107]. In a placebo-controlled trial, intravenously administered NAC had no effect on mortality or acidosis, and did not reduce erythrocyte rigidity in adults with SM [108]. Involvement of NAC in the metabolism of isoprostanes may have hampered is anti-oxidative effect [108]. Furthermore, as mentioned previously, the rapid action of intravenous artesunate might have blurred its clinical impact.

#### Correcting lactic acidosis

Metabolic acidosis is central to the pathophysiology of SM and is an independent predictor of fatality in both adults and children [109–112]. Dichloroacetate (DCA) stimulates pyruvate dehydrogenase activity and promotes the removal of pyruvate, the precursor of lactate. In an attempt to neutralize metabolic acidosis, DCA has been tested in small safety trials in children and adults. DCA was shown to reduce initial blood lactate levels, however, whether DCA will improve the outcome of SM remains to be seen [113–116].

#### Reduced nitric oxide bioavailability

Nitric oxide (NO) is produced from l-arginine and molecular oxygen by members of the nitric oxide synthase (NOS) family [117]. Limited NO levels can contribute to a number of pathophysiological processes involved in SM, including activation of the endothelium, stimulation of Weibel-Palade-body exocytosis, and increasing the expression of endothelial adhesion molecules (ICAM-1 and VCAM-1) [118, 119]. The use of inhaled NO (iNO) for the treatment of SM in children has been investigated in two RCTs. Both studies used iNO, administered at 80 parts per million for 48–72 h and both studies used markers of endothelial activation as their primary endpoints, namely the rate of decrease of Angiopoietin-2 (Ang-2), or the rate of increase in Angiopoietin-1(Ang-1) [120, 121]. Both studies found administration of iNO to be safe, but did not observe differences in circulating levels of Ang-1 and Ang-2 between treatment arms. It is possible that the dose and/or route of administration of NO was unable to cause a measurable effect on the endothelium or perhaps it is more suitable in the treatment of patients with increased cerebrovascular resistance [120, 121]. Alternative methods to increase NO levels, such as increasing plasma l-arginine levels via intravenous administration or increasing the bioavailability of cofactors required for NOS activity remain plausible interventions for adjunctive treatments [122–124].

### Novel strategies for adjunctive therapy delivery (preclinical murine models)

Animal models remain an useful tool to investigate novel adjunctive therapy [13]. Despite the large volume of research in experimental murine models, this discussion will be limited to preclinical studies where improvements have been observed in relation to novel treatments administered at the onset of clinical symptoms in ECM, and exclude studies of prophylactic treatment. This probably best resembles a clinical scenario where patients with severe disease seek treatment. Studies where adjunctive interventions have shown to protect against ECM-induced neurocognitive impairment will also be discussed (Table 2).

**Table 2:** Adjunctive therapy administered after the onset of neurological symptoms of ECM

Author, year, reference	Adjuvant Therapy	Route of administration	Outcome of treatment administered after neurological symptoms
Inmunomodulation			
Waknine-Grinberg et al. 2013, [124]	Glucocorticosteroids in liposomes	i.v. injection	Improved survival, prevented ECM symptoms, improved clinical scores
Dende et al. 2015, [127]	Curcumin	oral gavage	Improved survival, reduced parasitemia
Neuroprotection			
Dai et al. 2012, [129]	Lithium chloride	injection (route not described)	Prevention of cognitive and motor deficits. Reduced long-term motor coordination impairment. No effect on survival or parasitemia
Cabrales et al. 2010, [130]	Nimodipine	i.p. injection	Improved survival, improved motor score, reduced pial vasoconstriction
*Martins* et al. 2013, [132]	Nimodipine	s.c. osmotic pumps	Improved survival, reduced BBB dysfunction, reduced inflammation
Delivering gaseous signaling			
Orjuela-Sanchez et al. 2013, [133]	Glyceryl trinitrate	Transdermal patch	Improved survival, reversal of pial arteriolar vasoconstriction
Improving endothelial function			
Higgins et al. 2016 [140]	Recombinant human Ang-1	s.c. injection	Improved survival, prevents worsening of clinical outcomes, reduced cerebrovascular leak
Wilson et al. 2013, [141]	Atorvastatin	i.p. injection	Improved survival, reduced systemic and cerebral inflammation, reduces endothelial activation and reduced cerebrovascular leak
Dwivedi H et al. 2016, [145]	Vitamin D	i.m. injection	Improved survival, reduced cerebrovascular leak, reduced inflammation

*CQ* chloroquine, *ECM* experimental cerebral malaria, *i.m.* intramuscular, *IV* intravenous, *NO* nitric oxide, *s.c.* subcutaneous, *SM* severe malaria, *UM* uncomplicated malaria

#### Immunomodulation

New strategies to modify the immune response and target different pathways are ongoing. A recent study in ECM tested a new formulation of glucocorticosteroid, whereby β-methasone hemisuccinate (BMS) was encapsulated in lipososomes. Encapsulated BMS was less toxic to mice than the unencapsulated drug, and when administered at a late stage of infection it improved survival and prevented the development and progression of the cerebral syndrome [125]. These preclinical studies may lead to the use of new steroids for the treatment of SM.

Curcumin is an anti-inflammatory molecule that scavenges reactive oxygen and nitrogen species [126]. In vitro studies have shown that curcumin has additive anti-parasitic activity when used in combination with artemisinins [127]. When administered in combination with arteether to mice showing symptoms of CM, curcumin improved survival and prevented death due to anaemia [128].

#### Neuroprotection

Preclinical models have investigated lithium as a potential neuroprotective intervention. Lithium has been proposed to act as a neuroprotective agent by its ability to inhibit glycogen synthase kinase 3 (GSK3β), activate the PI3 K/Akt and MAPK signalling pathways, and by inducing the expression of brain-derived neurotrophic factors in neurons [129]. Lithium chloride administered to mice with ECM significantly increased the activation of Akt, which was associated with the prevention of adverse neurocognitive outcomes. Adjunctive treatment with lithium chloride was associated with better spatial and visual memory, and motor coordination in mice recovering from ECM [130].

Nimodipine is a calcium channel blocker that has been shown to prevent vasospasms, the abnormal physical narrowing of arteries in the sub-arachnoid space. Neuropathological features of CM include haemorrhages in the brain parenchyma [8]. It has been reported that mice with ECM show vasoconstriction and blood flow changes in the pia matter of the brain. Adjunctive treatment with nimodipine, when administered during late-stage infection, improved survival and improved blood flow to the brain [131]. However, potential hazards, such as hypotension, bradycardia and death can occur in humans treated with high doses of nimodipine [132]. Experiments have shown that in ECM, slow continuous administration of adjunctive nimodipine did not increase hypotension [133]. Additional preclinical work is required to determine if nimodipine is an attractive candidate as adjunctive therapy in SM.

#### Delivering gaseous signalling molecules

Increasing bioavailable NO in CM remains an attractive treatment strategy. A transdermal nitroglycerin patch was tested as an adjunctive therapy in late-stage ECM, where it increased plasma nitrate and nitrite levels (with no effect on blood pressure), and was associated with improved survival [134]. Haem oxygenase-1 (HO-1) catalyzes the degradation of haem and its activity has been shown to protect mice from ECM [135]. Prophylactic inhalation of carbon monoxide (CO), an endproduct of this catalysis, prevents mice from developing ECM and malaria-associated acute lung injury [135, 136]. The toxicity of inhaled CO limits its clinical utility. However, CO-releasing molecules that can deliver controlled amounts of CO to tissues are valid alternatives [137]. The CO-releasing molecule ALF492 significantly improved survival in ECM when administered with artesunate beyond the anti-malarial alone and without affecting oxygen transport by haemoglobin [138].

#### Improving endothelial function

Targeting endothelial activation and preventing microvascular permeability and vascular leak in CM is another potential target for adjunctive therapy [139]. The angiopoietin (Ang)-Tie2 axis critically regulates endothelial cell function [140]. Perturbation of Ang-1, Ang-2 and soluble Tie2 concentrations are associated with disease severity and death in CM in both murine models and human infections [141]. A mechanistic role for the Ang-Tie2 axis was established in ECM, where it was shown that Ang-1-deficient mice were more susceptible to ECM and adjunctive administration of a recombinant Ang-1 construct preserved BBB integrity and improved survival beyond artesunate monotherapy alone [141]. These studies provide preclinical evidence that interventions that target the Ang-Tie2 axis are potential adjunctive therapy for SM.

Atorvastatin, a drug that reduces cholesterol levels, also inhibits the expression of CXCL10, high levels of which have been associated with CM mortality in adult patients [142]. Mice deficient in CXCL10 are partially protected against ECM [143] and mice receiving atorvastatin treatment in addition to artemether upon neurological signs of ECM had improved survival, and increased transcription of Ang-1 and reduced levels of Ang-2 in brain tissues [144].

Vitamin D may improve survival by targeting multiple pathways in both the innate and acquired immune systems [145]. One study showed that simultaneous administration of intramuscular arteether and vitamin D to mice at the onset of neurological symptoms of ECM improved survival. This survival was accompanied by reduced BBB leak and reduced levels of circulating pro-inflammatory cytokines [146].

Inhibition of the angiotensin pathway is another strategy to maintain endothelial integrity by preserving inter-endothelial cell junctions. Blocking the angiotensin II type 1 receptor with Irbesartan or activation of the type 2 receptor with compound 21 in combination with chloroquine resulted in an increased survival rate, higher than when treated with the anti-malarial alone, even when mice were treated at the onset of neurological symptoms [147].

## Conclusions

Malaria remains a major global health problem, associated with high morbidity and mortality. Strategies designed to improve early detection and recognition of cases likely to progress towards to severe disease, so as to trigger immediate treatment, are absolutely necessary. For those individuals who progress to severe forms of the disease despite prompt treatment, new tools are needed to improve outcomes in addition to existing anti-malarials. Preventing long-term *sequelae*, such as improving neurocognitive outcomes in SM survivors, should be an important consideration when it comes to potential adjunctive therapy; however so far, the majority of attempts to enhance the efficacy of anti-malarial drugs with adjunctive therapy have failed. The development of adjunctive therapy would benefit from a more complete understanding of the physiopathology of SM and CM, and how it differs between adults and children. The identification of host biomarkers associated with disease severity and host response to treatment could provide a useful read out of therapeutic efficacy, and empower RCTs to evaluate adjunctive therapy with smaller and better defined cohorts. Therapy tested in preclinical models of SM are still a valuable resource for potential adjunctive therapy; however preclinical models should employ scenarios as similar as possible to clinical practice, targeting the onset of clinical disease symptoms and prevention of long-term *sequelae*. RCTs in humans should also be guided by a rational and good design based on well-defined sample sizes, clinical predictors and study endpoints that permit detection of significant differences in SM outcomes and direct comparison between studies. It is difficult to extrapolate conclusions and conceive future research considering the heterogeneity of the RCTs in terms of anti-malarial used, type of malaria (SM and/or CM, coma), or study characteristics (limited number of patients per study, different and no comparable age of the populations, different treatment doses, studies not designed to identify differences in clinical outcomes or mortality). Further research, with promising candidates that surpass previous constraints of earlier studies, is urgently needed in order to accelerate the identification of new adjunctive therapy for the treatment of SM.

## Abbreviations

ADHD: attention-deficit hyperactivity disorder
ALT: alanine aminotransferase
Ang: angiopoietin
Ang-1: angiopoietin-1
Ang-2: angiopoietin-2
AST: aspartate aminotransferase
BBB: blood–brain-barrier
BDNF: brain-derived neurotrophic factor
BMS: β-methasone hemisuccinate
CFR: case fatality rate
CM: cerebral malaria
CO: carbon monoxide
CS: curdlan sulfate
CT: computed tomography
DCA: dichloroacetate
DFO: desferrioxamine
EBT: exchange blood transfusions
ECM: experimental cerebral malaria
EPCR: endothelial protein C receptor
GAG: sulfated glycosaminoglycans
GSK3β: glycogen synthase kinase 3
HIV: human immunodeficiency virus
HO-1: haem oxygenase-1
iNO: inhaled nitric oxide
iRBC: infected red blood cells
MRI: magnetic resonance imaging
NAC: N-acetylcysteine
NO: nitric oxide
oAc: activated charcoal
PEs: parasitized erythrocytes
PfEMP1: *Plasmodium falciparum* erythrocyte membrane protein 1
PPAR-γ: peroxisome proliferator-activated receptor-γ
RCT: randomized controlled trial
rHuEPO: recombinant human erythropoietin
SM: severe malaria
SMA: severe malarial anemia
TNF: tumour necrosis factor
PTX: pentoxifylline
oAC: oral activated charcoal
UDCA: ursodeoxycholic acid
